# ‘Maybe I will give some help…. maybe not to help the eyes but different help’: an analysis of care and support of children with visual impairment in community settings in Malawi

**DOI:** 10.1111/cch.12462

**Published:** 2017-04-09

**Authors:** M. Gladstone, M. McLinden, G. Douglas, E. Jolley, E. Schmidt, J. Chimoyo, H. Magombo, P. Lynch

**Affiliations:** ^1^ Department of Women and Children's Health, Institute of Translational MedicineUniversity of Liverpool, Alder Hey NHS Children's Foundation TrustLiverpoolUK; ^2^ Visual Impairment Centre for Teaching and Research (VICTAR), School of EducationUniversity of BirminghamBirminghamUK; ^3^ Department of Strategic Programme DevelopmentEvidence and Research (SPIDER)SightsaversWest SussexUK; ^4^ Montfort Special Needs Education CollegeLimbeMalawi

**Keywords:** Africa, child disability, early child development, low and middle income, qualitative, visual impairment

## Abstract

**Background:**

Visual impairment in children is common in low and middle‐income settings. Whilst visual impairment (VI) can impact on the development of children, many reach full potential with appropriate early intervention programmes. Although there is increased emphasis on early child development globally, it is not yet clear how to provide specific programmes for children with VI in low and middle‐income settings. This study aims to identify facilitators and barriers to the provision of a developmental stimulation programme for children with VI in rural and urban Malawi.

**Methods:**

We undertook 6 focus groups, 10 home observations and 20 in‐depth interviews with carers of children with VI under 6 years in urban and rural Southern Malawi. We utilised topic guides relating to care, play, communication and feeding. Qualitative data were subject to thematic analysis that included placing themes within Bronfenbrenner's ecological framework. We established authenticity of themes through feedback from participants.

**Results:**

We identified themes within Bronfenbrenner's framework at five levels: (1) blindness acting as a barrier to stimulation and communication, health and complex needs all affecting the individual child; (2) understanding of VI, ability to be responsive at the microsystem level of the carer; (3) support from other carers at microsystem level within a mesosystem; (4) support from other professionals (knowledge of, identification and management of children with VI, responsibilities and gender roles, environmental safety and prejudice, stigma and child protection all at the level of the exosystem.

**Discussion:**

This study has revealed the requirements needed in order to produce meaningful and appropriate programmes to support nutrition, care and early stimulation for children with VI in this and similar African settings. This includes supporting carers to understand their child's developmental needs, how to better communicate with, feed and stimulate their child; offering advice sensitive to carers' responsibilities and professional training to better support carers and challenge community stigma.

## Introduction

The number of children aged 0–14 years living with moderate or severe disability globally is estimated at 93 million (UNICEF 2013) with 19 million (20%) of those visually impaired (World Health Organization 2014).

The high rates of developmental delay in children in low and middle‐income (LMIC) setting are a current major concern of the global community (McCoy *et al.* 2016). Factors contributing to this are multiple, and there is consensus that investing in programmes that encourage developmental stimulation and responsiveness can improve outcomes for these children (Aboud & Yousafzai 2015) particularly in the first 1000 days of life (Black *et al.* 2016). International agencies such as WHO and UNICEF have placed increased emphasis on developing and promoting equity‐based approaches to ensure that early interventions are inclusive of all children including those with disabilities. Whilst there are potential barriers to access and participation for these children, research demonstrates the importance of ensuring timely interventions, particularly for those with visual impairment (VI) (Webster & Roe 1998; Dale & Sonksen 2002). Vision, being the ‘coordinating sense’ is important in developing basic sensorimotor understanding (Brambring 2007), early social behaviour and communication (Troster *et al.* 1992). Early disruption to this through lack of early eye contact and joint referencing can lead to developmental difficulties. Interventions for children with VI which encourage them to build on their existing visual abilities as well as other senses is crucial in enabling these children to do better (Ferrell 1996). Evidence from UK demonstrates that visually impaired children can significantly benefit from stimulation, and that lack of appropriate stimulation can result in developmental delay (Cass *et al.* 1994; Dale & Sonksen 2002). Such studies have led to the development of programmes that provide information on how to support these children. Programmes include the Oregon Project (Anderson *et al.* 2007), the Simmons Davidson Developmental Profile (Simmons & Davidson 1992) and the Developmental Journal (Dale & Salt 2010). There is no evidence of the efficacy of similar programmes in Africa or other LMIC settings, or whether these programmes could be adapted where infrastructure is limited. This, we need to know before we can attempt to embed programmes in other cultural and ecological contexts (World Health Organization and UNICEF 2012).

The Ecological Systems framework conceptualised by Bronfenbrenner provides a suitable lens through which to examine these contexts. Bronfenbrenner's framework promotes the idea that human development takes place through processes of reciprocal interaction between the developing child and the persons, objects and symbols in the environment. The form, power, content and direction of these processes vary as a joint function of the characteristics of the developing child, of the environment (both immediate and remote) in which the processes are taking place and the nature of the developmental outcomes under consideration. This is illustrated as a nested system of ‘environments’ which interplay with one another. These systems include; the ‘microsystem’ – the interpersonal relationship and physical setting directly experienced by the child (family, peer group, school and neighbourhood), the ‘mesosystem’ – the linkages and processes taking place between two or more microsystems (e.g. school and home) the ‘exosystem’ – the linkages and processes taking place between home and settings which may indirectly influence processes in the immediate setting (e.g. parent workplace, family and social networks and neighbourhood and community contexts) and finally, the macrosystem – the overarching pattern of the micro, meso and exosystems within a given culture and setting (e.g. policies, structural factors, life course options and resources) (Bronfenbrenner 1994).

This framework has influenced researchers examining the interplay of the family group ((Whiting 1975; Rogoff 1990; Levine *et al.* 1994), community and wider society (Lancy 2010) on child development within cultural contexts. Some studies have considered how ecological factors may affect the care of the child and furthermore, how this may influence the impact of developmental stimulation programmes (Aboud & Alemu 1995; Aboud 2007; Lingam *et al.* 2014). Very few studies have specifically studied factors that affect the care, stimulation and play of children with disabilities. Other studies have concentrated on caregiver burden such as physical pain from handling children with disabilities, time taken for daily care, financial strain and stigmatisation from communities and parental stress (Gona *et al.* 2011; Mushi *et al.* 2012; Geere *et al.* 2013). These studies identify factors which can serve as potential ‘barriers’ and ‘facilitator's to developmental progression as well as activity and participation for children with any disability. There are presently no studies examining the barriers and facilitators to care, specifically for children with visual impairment particularly taking into account early developmental care practices (play, stimulation and interaction) for these children in LMIC settings.

The purpose of this study was to understand and contextualise the experiences and perspectives of parent/carers (PCs) of pre‐school children with visual impairment. Our aim was to identify ‘facilitators’ and ‘barriers’ to enable the development of an appropriate training package to encourage stimulation for children with VI by community workers in rural and urban areas of Malawi.

## Methods

We undertook a qualitative study to investigate the personal experience, knowledge and understanding of PCs looking after children with VI (Pope & Mays 1995; Kielmann *et al.* 2011). We utilised a variety of approaches to understand perspectives, beliefs, and attitudes and lived experiences individually, through in‐depth interviews (IDI) (Britten 1995) and shared, through focus group discussions (FGDs). This enabled credibility and confirmability, between different perspectives (Kitzinger 1995). We also used observational methods to generate a description of ‘days in the life’ of young children with VI, which highlighted (i) parent (carer)/child interactions; (ii) contexts and patterns of behaviour; and (iii) opportunities for interaction. For observations, we developed a schedule based on a semi‐structured interval‐sampling approach to generate descriptions of interactions in 1‐min bands (Sylva *et al.* 1980). The approach was naturalistic within the normal routine of the parent and child's day. The observer was required to provide contextual information and initial interpretation to the observation (Bannister *et al.* 1994).

### Study setting and participant selection

The study took place in Southern Malawi in rural (Chikwawa and Blantyre) and urban Blantyre from March 2013–March 2014. The project was built on an existing early childhood development and disability project where children with disabilities (including visual impairment) were previously identified (Sightsavers 2012).

There has been an increasing investment in early child development through the creation of CBCCs which are now almost universal in Malawi. Despite this, children with disabilities in Malawi have limited services, and very few attend a CBCC (Neuman *et al.* 2014). A handful of specialised services exist in urban settings. Some have links with poorly trained Community Based Rehabilitation (CBR) workers or community health staff and few see an itinerant teacher (Paget *et al.* 2016).

We utilised purposive sampling to enable recruitment of a range of PCs with children with VI (0–6 years). We recruited participants using registers from itinerant teachers, community workers and ophthalmology units. We provided Information on the study through this network, and those interested were contacted for consent. Table 1 shows a list of participants and their defining features. Sampling continued until data saturation was met and no new codes were developed.

**Table 1:** Characteristics of individuals and groups in interviews, observations and focus groups

ID number	Location	Age of interviewee's child (years)	Gender of interviewee's child	Diagnosis of interviewee's child	Diagnosis of interviewee's child
VICD_IDI_1	Blantyre Urban	3	F	Epilepsy developmental delay	Blind
VICD_IDI_2	Blantyre Urban	1.5	M	Cerebral palsy	Blind
VICD_IDI_3	Blantyre Urban	5	F	Congenital blindness	Blind
VICD_IDI_4	Blantyre Urban	2.5	M	Glaucoma	Low vision
VICD_IDI_5	Blantyre Urban	6	M	Albinism	Low vision
VICD_IDI_6	Blantyre Urban	6	F	Albinism	Low vision
VICD_IDI_7	Blantyre Rural	3	F	Congenital blindness	Blind
VICD_IDI_8	Blantyre Rural	3	M	Exact diagnosis not known	Low vision
VICD_IDI_9	Blantyre Rural	5	M	Exact diagnosis not known	Low vision
VICD_IDI_10	Blantyre Rural	5	M	Exact diagnosis not known	Low vision
VICD_IDI_11	Blantyre Rural	3.5	M	Exact diagnosis not known	Low vision
VICD_IDI_12	Blantyre Rural	3	M	Congenital problem	Low vision
VICD_IDI_13	Blantyre Rural	5	M	Exact diagnosis not known	Blind
VICD_IDI_14	Chikwawa	1	F	Albinism	Low vision
VICD_IDI_15	Chikwawa	6	F	Hearing and vision problems	Low vision
VICD_IDI_16	Chikwawa	5	F	Exact diagnosis not known	Low vision
VICD_IDI_17	Chikwawa	2	M	Exact diagnosis not known	Low vision
VICD_IDI_18	Chikwawa	6	F	Exact diagnosis not known	Low vision
VICD_IDI_19	Chikwawa	5	F	Exact diagnosis not known	Low vision
VICD_IDI_20	Chikwawa	4	M	Exact diagnosis not known	Low vision
Observations:					
VICD_Obs_1	Blantyre Urban	1.5	F	Epilepsy and genetic condition	Blind,
VICD_Obs_2	Blantyre Urban	1.5	M	Cerebral palsy	Blind
VICD_Obs_3	Blantyre Urban	5	M	Glaucoma	Blind
VICD_Obs_4	Blantyre Rural	1.5	M	Congenital blindness	Blind
VICD_Obs_5	Blantyre Rural	3	M	Albinism	Low vision
VICD_Obs_6	Blantyre Rural	0.8	M	Albinism	Low vision
VICD_Obs_7	Chikwawa	3	M	Multiple disabilities	Blind
VICD_Obs_8	Chikwawa	2.5	M	Albinism	Low vision
VICD_Obs_9	Chikwawa	5	M	Epilepsy	Low vision
VICD_Obs_10	Chikwawa	5	F	Exact diagnosis not known	Low vision
Focus groups					
Focus group	Location			Number in group	
VICD_FGD_1	Blantyre Urban			3	
VICD_FGD_2	Blantyre Urban			3	
VICD_FGD_3	Blantyre Rural			4	
VICD_FGD_4	Blantyre Rural			4	
VICD_FGD_5	Chikwawa			4	
VICD_FGD_6	Chikwawa			4	

## Data collection, management and analysis

In‐depth interviews and FGDs were conducted and audio‐recorded by the research assistant (J. C.) with support from M. G. and E. N. in the early stages. Transcripts were checked for quality and analysed using QSR NVIVO 10. The research coordinator (H. M.) and research assistant (J. C.) were both from the Southern Malawi and speak Chichewa fluently. J. C. and H. M. are both in their mid 30s and had training in special needs support. Interviews and FGDs took place in Chichewa, the main language spoken in Southern Malawi, and the data were transcribed and translated by two transcribers to enable quality control. Observations were conducted by J. C. over 4‐h periods at different times of day enabling capture of different events in the data. J. C. was provided with training by P. L. and E. N. Both E. J. and J. C. carried out three observations of the same child at the same time, which was followed up by discussions and agreement on how much time to observe parent–child at home and how much time to record the observation. M. G. is a white female doctor from the UK who provided external advice and support. P. L. and E. N. were white research fellow in special needs from the UK who also provided external advice and support.

**Figure 1:**
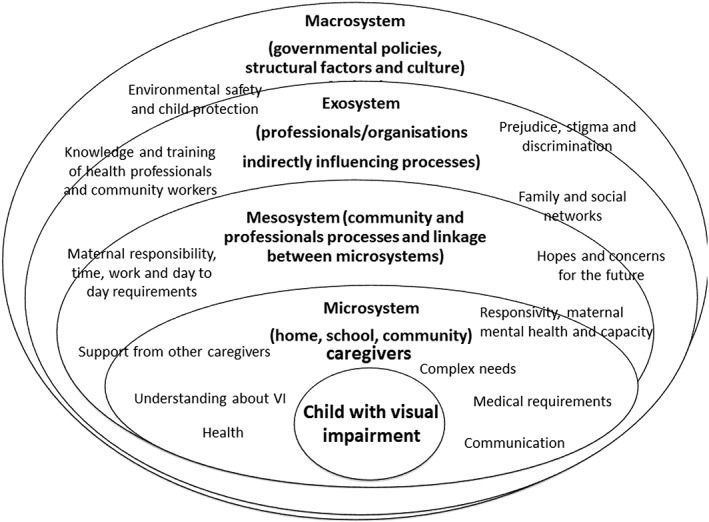
Diagram showing levels at which there are facilitators and barriers to the care for the child with visual impairment

Thematic content analysis used an inductive approach where themes were coded as they emerged from data. A list of 47 free codes was created by four members of the research team (M. G., P. L., E. N. and M. K.), which eventually fell into 33 themes. Authors coded transcripts and compared codes electronically and then in a consensus meeting prior to the final codes being decided on. We reviewed and restructured these through consensus and expanded coding when necessary in some areas through continuous review and updating to reflect emerging ideas (Pope *et al.* 2000). Codes were collapsed into a coding framework with main themes and sub‐themes under each of Bronfenbrenner's hierarchical levels and placed within the framework (Fig. 1) (Bronfenbrenner 1996; Jennings *et al.* 2009).

Following analysis, authenticity was further established utilising feedback from participants (carers of children with VI and associated professionals) to triangulate results (Kuper *et al.* 2008). A team of professionals and carers caring for children with VI were involved in a steering group throughout the research to provide advice on appropriate methodology, topic guides and processes for conducting the research. This team enabled us to disseminate our results at early stages to confirm a representative understanding of emerging themes and findings. We held research dissemination meetings at a Specialist teacher training college (Montfort College) and at a stakeholders' meeting for respondent validation.

### Ethics

Ethical approval was provided by College of Medicine Research Ethics Committee (P.11/12/1307) and University of Birmingham research ethics committee (ERN_12–0916). All potential participants received information in English and Chichewa about the purpose and procedures of the study, in written and verbal formats. A written consent form was completed and verbal consent audio recorded before commencing.

## Results

### Sample of children and carers selected

We conducted 20 IDI, 10 observations and 6 FGDs with carers/carers of children with VI (Table 1). A total of 15 children had low vision, and five were blind. One child had cerebral palsy, one had epilepsy and developmental delay, four had oculo‐cutaneous albinism, and some had no clear diagnosis.

### Thematic framework

We report on facilitators and barriers that we identified in relation to each concentric level of Bronfenbrener's ecological framework (Fig. 1) at (1) the level of the child (individual); (2) the carer and family (microsystem); (3) the community (microsystem and mesosystem); (4) the teacher and other professional organisations (mesosystem and exosystem); and finally, (5) the level of policy and culture (exosystem). We also contextualise themes and results in relation to barriers and facilitators to enabling better care, early stimulation and communication with children with VI.

1 The child (individual) with visual impairment and the direct impact on early child development and play

Specific issues relating to the impact of the visual impairment on the individual child's capacity to play or interact emerged. It is clear that the needs of children are variable depending on their underlying disability or health condition.

#### ‘When the child cannot see there is no stimulus to play’.

Visual impairment directly impacts on the child's ability to communicate, play and build important concepts of the social and physical world. A lack of visual, tactile or auditory stimuli can seriously affect their motivation to play. Some carers found it difficult to play with their children with VI and also did not know that their children could receive stimulation through other sensory modalities (touch, smell and sound).

*‘When the child cannot see there is no stimulus for him to play … I am saying this with reference to my child …. He does not have motivation to play that is why I am finding it hard …. I… doubt whether my child will ever play [Seeming frustrated] because he cannot see’ (VICD_FGD3).*

#### ‘When children are playing, it means they are ok health‐wise’.

The ability for children to enter into play is intimately bound with wellness according to many carers. When carers were asked about play, many referred to the child who played as the healthy child.

*‘children's play means that the child's body parts are able to work properly and on their own without…difficulties.’(VICD_IDI_12).*

Carers did not describe play as something that you ‘do with your child’ but something that a child does when they are well;

*‘most of the times she likes to play or do something else, she doesn't remain idle unless she is sick, she is full of life.’ (VICD_IDI_18).*

#### ‘Sometimes he feels pain in his eyes’ – medical requirements for children with visual impairment

Some carers described specific difficulties with their children's eyes and explained how this impinged on child's play. Itchy or painful eyes sometimes stop children from wanting to play; ‘*My child is always on my back because of the eye problem but when he is not, then what he does is to scratch it continuously and he would want me to do it as well’ (VICD_IDI_8).* Some children with albinism experience difficulties with glare; ‘*the other problem with eyes is the sun, when the sun shines so bright the eyes ache, when it is cold the people who have eye problems feel better... (VICD_FGD5)’.*

Other issues included*; eye mucus* ‘*In the morning s/he wakes up with a lot of eye mucus……..sometimes s/he can't see properly, doesn't walk straight’ (VICDFGD5).*

#### ‘I find it difficult to know what she is crying for’: Difficulties with communication and behaviour for children with visual impairment

Many carers described difficulties in managing behaviour in children with visual impairment particularly disruptive and obstructive behaviour. Often a lack of communication underpins a child's delayed social and emotional development. One mother explained how; ‘*when I am communicating with him he doesn't want to listen to what I am telling him…..he makes sure that I must give him whatever he wants or otherwise he cries uncontrollably…sometimes I have no choice than to search for whatever he wants just to make him happy…..’ (VICD_IDI_5)* Another mother explained how it was not until she understood her child's non‐verbal communication, that she understood her child*; ‘She spits it out when I try feeding her and I realized she wants to eat on her own but wants me just to put it into her hand so I just put it in a plate and she eats from there’ (VICD_FGD_3).*

#### ‘After seeing him/her, he maybe will give some help…. Maybe not to help the eyes but different help’. Complex needs of children with visual impairment

All children in this study had VI, but a number had other medical needs or impairments. These children need support from other services. One mother explains how some services only concentrate on the other impairments ‘*this problem…it is in his/her body …..l see that the real help they are not giving me so l just stopped..The problem with the eyes is there but they do not help me with anything, l just got tired of walking’ (VICD_FGD5).* Another mum explained the multiple needs of her child*; ‘she has a hearing problem as well as eye problems because when you ask her to do something else, she does different things and not what you have asked her to do’.(VICD_IDI_15)*

2 Microsystems (the carer and family and other carers in the community) within a larger mesosystem

### The carer and child

#### The carer's understanding about visual impairment and how to identify that children have a problem.

The need to know more about their child's condition is paramount. Many carers described how difficult it was to get advice from health professionals. Most carers did not know that their child could play, grow or develop in the same way as other children if given the right support or intervention. Carers discussed ways that they thought they could tell whether their child had a problem with their eyes; ‘*We expect to know if the child has sight problems by waving a hand in the child's face and observing, is the child following the waving hand… That is when we would know if the child can see or not’ (VICD_FGD4); ‘there are some when they are looking at a thing their eyes move quickly, so we know that s/he has eye problems’(VICD_FGD5).*

#### Responsivity and effective early communication by carers ‘I have to hold her to say I am here with you I am not going anywhere’

Carers clearly desire and demonstrate responsive behaviour to their children, adapting their communication and interaction to their child, including using touch and audio‐description; ‘*the child is already sad thinking that the hand was deliberately withdrawn because he cannot see, but you make sure you put the hand back again and when the child feels it, laughs and is assured he is still with the mother’(VICD_FGD3).* One mum describes the close bond she has with her child and how she *‘feels’ what her child wants her to do; ‘I feel like she wants me to play with her and I reciprocate by touching her as well’ (VICD_IDI_1).* One carer described back and forth play and was proud of her knowledge of the child's needs*; ‘I put those things in‐front of her, in between her legs and she touches those things and when it disappears I bring it back for her or sometimes she searches it using her right hand’(VICD_IDI_7)*. Another mother clearly describes her awareness of her child responding and communicating through movement; *‘when I am passing by she touches my clothes and I reciprocate and you will see her smiling and laughing feeling good about it’(VICD_IDI_7)*.

Although carers describe stimulating and interacting with their children with VI, ‘talking to your baby’ for the sake of it does not feature. Carers describe chatting to their children pragmatically, but not specifically to stimulate them; ‘*when he is awake I first of all talk to her by greeting her so that she should be able to know that I am around her.’ (VICD_IDI_1)*. No mother mentioned talking as a form of stimulation, but many mums described or were seen singing with their babies. Incidentally, this creates an important sense of ‘presence’ for the baby who will not be able to depend on visual cues (e.g. seeing mother in the same room).

Fathers feature in care of children more so in urban settings but less commonly than mothers. Fathers were more likely to be involved by default; ‘*when her father is around, he plays with her by making some rolling with her more especially when she is on her bed in our bedroom, sometimes he helps her to stand using his hand and plays with her.’(VICD_IDI1)*; ‘*when I am not around, he cooks…and gives him a bath and makes sure that he takes his share of food’ (VICD_IDI13)*; ‘*my husband is there to support us but then it's mostly me who does those things’ (VICD_IDI13).*

There were only a few descriptions or observations of intense one to one verbal exchange between carers and their children. Interactions that were observed consisted of clapping or singing with the child; ‘*child starts to shout Aaa! Aaa! Taa! Taa!... She holds the child's hands together and helps to clap her hands too’ (VICD Obs4); ‘The mother starts to sing some songs for her; ‘gona mwana, uleke kulira, mbalame zonse zagona, muzisa zawo zazing'ono, gona mwana’, (Sleep my child, stop crying, all the birds are sleeping in their small nest, so sleep my child.’(VICD Obs4).* Some carers narrate the daily routine to help their children make sense of the social and physical world; ‘*I first of all inform her that she is going to take a bath, then I remove her clothes and she is able to understand that she is going to take a bath.’(*VICD_IDI_1)

#### Hopes and concerns for the future

Some carers describe their concern about how children cope in the outside world; *‘When you do not have eyes to see …. you cannot freely perform some tasks and your body…is filled with worries…we as carers….also wonder why God allowed that to befall us.’ (VICD_IDI4).*

Many carers discussed a lack of hope for the future education of their children. Some described how helpful an early child care centre would be; ’*that school (nursery) is a nice and good place because I see some of the care givers [volunteers who work in the CBCCs] doing some exercises with some children who are disabled, they actually know what to do with any type of disability….’(VICD_IDI1).*

### Community carers and the child

#### Support from other carers: ‘it means that his friends are trying to make him happy because of his eye problem’

Many families receive support from immediate family and distant relatives, friends and neighbours. Children with VI are often included in games of siblings and neighbours; ‘*They play with her when they find her at home, they come and play with her. When they play with her we tell them not to play with her in the sand……they play well with her, hold her and when playing watch her.. (VICD_FGD5)’.*

3 The Exosystem (professionals/organisations indirectly influencing processes)

#### Support from teachers and professionals at community and facility level and their linkage to parents and families;

A number of families described going from one health centre to another to try to get support for little benefit in what professionals were able to provide for them. Some urban families managed to get advice from FEDOMA (organisation representing disabled persons) or from specialist teachers for VI (itinerant teachers) but no families in rural settings mentioned getting specific advice about how to care for their child; ‘*you take him to the hospital. When you see that at that hospital are not helping, you go to another hospital…. then the doctor should fail himself’ (Chikwawa FGD PC)* .

4 The Mesosystem (governmental policies, structural factors and culture)

#### Prejudice/stigma – ‘in our homes let us love all children and let us love more the one with the physically challenged one so that he has a healthy life’

Many carers describe the stigma that comes with having a child with a disability; ‘*She is saying you should tell them that they are no better than her and she is as capable as they are. She has…rights’ (VICD_IDI4).* There is often a lack of acceptance of children with VI with some children even harmed by others in the community. One mum explains how much of a difference it makes when her child is accepted; ‘*if there are good people it works…. but if not, then it doesn't work for him’ (VICD_IDI9).*

#### Maternal responsibility, time and day to day requirements: ‘during sunrise I prepared fire, swept the surrounding, put water on the fire, bathed her while the porridge was on the fire’.

The burden of daily routines and responsibilities was one of the most prominent themes for all carers (all women and mostly mothers); ‘*I always make sure that I provide food for my child when she is awake. I give her porridge then after eating she takes a bath, …. while she is resting, I should be able to finish my…chores then after that, I play with her and give her…exercise’ (VICD‐IDI_1).*

The constant requirements for some families to search for food means that playing comes further down the list of priorities; ‘*I leave him on the mat in the morning with his friends when I am gone to the field, so I do not know what time he wakes up’(VICD_IDI12).* Many carers described the extra need to escort their children with VI to school or nursery, something less needed for a ‘seeing’ child. One mother describes the constant demands of supporting a child with multiple impairments; ‘*I have to pick some toys for her when she has lost it because it is difficult for her to…reach it and that disturbs my work at home… it means that I have to be there always for her’ (VICD_IDI_1).* Another mother describes her struggle with no support from the external community; *‘when I wake up in the morning I take water and clean her face, then after that she takes her breakfast and wherever I go, I put her on my back and off we go’. (VICD_IDI_14)*. We provide more examples of barriers and facilitators in Table 2.

**Table 2:** Summary table of barriers or enablers to supporting early child care and development for children with VI

Theme	Facilitators to play, communication and developmental stimulation	Barriers to play, communication and developmental stimulation (responsiveness)
Nutrition and health as a priority to play and communication	*‘It is like he has strength when playing’ ((VICD_IDI_1).* *when children are playing it means that they are okay health wise and ‘even the carers becomes happy to see them playing’ (VICD_IDI_13)*	*‘He is still searching for something to eat ….but it seems like he is still complaining of being hungry …but the mother did not respond may be because there is nothing to give to him at the moment.’ (VICD_OBS9)*
Multiple responsibilities of caregivers and involvement of other caregivers	*‘when her father is around, he plays with her by making some rolling with her more especially when she is on her bed in our bedroom, sometimes he helps her to stand using his hand and plays with her’. (VICD_IDI_1)*	*‘the mother ignores him at first and continues preparing the nuts. …….The mother is asking him to go and play with his friend’ (VICD Obs5)* *‘most of the times you cannot leave her alone, I always have to be there for her own security.when she is falling down I have to control her or pick her’ VICD_IDI_1).*
Playing on their own – dependency and independence	*‘when maybe it's a new thing, she will ask for help but when she is used to it, then she would play with it alone, so when I have to cook I give her building blocks then she plays with it on her own’ (VICD_IDI_3).*	*‘…it is difficult for her to find or reach it and that disturbs my work at home because it means that I have to be there always for her’ (VICD_IDI_1).*
Identification and understanding of visual impairment	*‘have been going to the hospital for so many times and during the last trip that was when I met the person who identified me and my child’ (VICD_IDI_9).*	*‘When I pass you may think s/he is seeing you but when you go back s/he never turns, so I see that ooh, s/he did not realize that someone has passed here yeah. So you keep wondering that so it means that the child is not seeing properly.’(VICD_FGD5).*
Use of play materials.	*‘we must provide playing materials which are so bright, so that if the child has low vision she should be able to see and identify it (VICD_IDI_1),* *if there are playing materials around the child then she/he is able to touch those things with her/his hands,’ (VICD_IDI_1),* *‘something like a comb.tie to it some bell,ring it for the child’ (VICD_FGD6)* *‘Or else we can find a tin and put stones into it and close it and give it to the child to play with it and it will make noise’ (VICD_FGD6)*	*‘I bought some at K500, some K200 and the other doll I bought it at K1000 which is very expensive’ (VICD_IDI_1) COST…* *‘We can get a cloth and sew to it biscuits, crisp wrappers to make it as it is. We can also continue thinking, can't we’ (VICD_FGD5)* *‘Some, pots, this, water. Even fire, when play cooking they make fire.’(VICD_FGD5)* *‘To play with it and s/he never allows to put it down, s/he clings to it may be up to when we leave’ (VICD_FGD5).*
Child being well and not in pain	*‘he likes to play with the ball by passing it to each other and if his eyes are fine, he plays it without any problems’ (VICD_IDI_11)*	*‘he finds it difficult to play or concentrate when playing because of the pain he experiences at that time’.(VICD_IDI_11)*
Other children	*‘his friends are the ones who take the ball and give it to him on his hands and then he throws it using the hand and sometimes using the leg and when it goes out of the ground’(VICD_IDI_13).* *‘she doesn't seek me but she seeks her friend's presence when they want to sing their songs, clapping their hands and dancing together while listening to the cassette’(VICD_IDI_16).*	*‘what I have noticed is that sometimes when they are playing, her friends mock her by saying a lot of things concerning the eye, and they sometimes tell her that her eye looks different from the normal eye’ (VICD_IDI_18).* *‘he does not do anything because he can't speak, he just look at them without saying a word. I see him; I do observe him that he wishes he could have joined his friends who are playing at a distance’ (VICD_IDI_2).*
Multiple needs, additional complex needs	*‘so when l told the MAP (Malawi Against Physical Disabilities) that the child does not see properly how are you going to help me so they say ah we should finish with this problem…in his/her body ‘(VICD_FGD5).*	*‘It seems like she also has a hearing problem as well… I try my level best to explain things to her but it seems like she doesn't understand…that is the only thing that makes it hard when I am communicating with her’ (VICD_IDI_15).*
Communication	Facilitators	Barriers
Use of other senses	*‘Yes they touch her and sing her songs and she laughs.And some of her friends touch her. She laughs only when they touch her’ (VICD_FGD3)* *‘The child may not talk but touch your child and say how are you this morning my child while massaging him…so that he appreciates he is in his mother's arms. And call his name…’ (VICD_FGD3)*	***‘*** *While if s/he hasn't see the thing she can never bother to what? To see it’ (VICD_FGD5).* *‘When its others they can not see,they can not hear,so we do not communicate with him/her properly.’ (VICD_FGD5).*
Using communication to demonstrate routines	*‘We then take the porridge and start to feed him and at this moment he knows what is happening. I do not know whether it is through smelling. He knows that food is ready and he starts opening his mouth ‐ we tell him like we are telling a story, we inform him that we are about to give him something and we give him by holding the thing on his hand’ (VICD_IDI2).* *‘When we give the child the object that is to be used for playing…and explain what it is. so next time when we want to play he/ she lifts up there hands or time to play ball they now what it involves’ (VICD_FGD1).*	*‘Like when I inform her that it's time to eat I feel like she doesn't understand whatever I told her because sometimes when I give her the food she cries meaning that she did not listen to what I said even though I continue giving the food by forcing her and sometimes when I mentioned her name she becomes frightened and cries and that is the time I remember that she is blind’ (VICD_IDI_7).* *‘What brings the difference is the lack of sight which prevents the child from mentioning something he wants because he does not know what it is’ (VICD_FGD3).*
Ability to show emotions and behaviour	*‘She spits it out when I try feeding her and I realized she wants to eat on her own but wants me just to put it into her hand so I just put it in a plate and she eats from there’ (VICD_IDI_1)*	*‘The child's behaviour changes when he reaches the age of one year and six months or seven as he isolates himself from the rest of the people around him’(VICD_FGD2).* *‘To show his frustration he starts singing so loudly to one of the local songs and the mother stops him because according to her, he is making noise’ (VICD_Obs9).*
Routines	*‘sometimes she knows when I want to go out somewhere because she is used to whatever I do, she knows that the way my mother is doing today, she is going somewhere, that is also part of communication and again she also knows that today I am going to school because I put her on uniform, that is also part of communication even when you do not actually say that today she going to school but through actions’ (VICD_IDI_6).*	*‘like when I ask him to go and get something from either the kitchen, he finds it hard to find that particular thing even when… is near him because of the eye problem’* (VICD_IDI_4) *‘Like when I inform her that it's time to eat I feel like she doesn't understand whatever I told her because sometimes when I give her the food she cries meaning that she did not listen to what I said even though I continue giving the food by forcing her and sometimes when I mentioned her name she becomes frightened and cries and that is the time I remember that she is blind’ (VICD_IDI6).*
Developmental support and stimulation	Facilitators	Barriers
Support and mental health of caregiver	*‘My husband is there to support us’ (VICD_IDI_1).*	*‘Sometimes you are anxious or depressed when you are chatting with friends whose children can see’ (VICD_FGD3).*
Sense of innate understanding of the child	*‘yes.sometimes when I am passing. She touches my clothes and I feel like she wants me to play with her. I reciprocate by touching her as well and you will see her smiling and laughing feeling good about it’(VICD_IDI_7).*	*‘there are times when he disobeys when you are advising him not to do certain things, so I find it very hard for us to communicate properly so that he may understand what I am trying to say or what it is supposed to be done’* (VICD_IDI_4).
Need for mum to be mindful of the child	*‘It all takes a mum's deliberate effort to study her child in order to discern what the child precisely wants... This is different from someone who can see who can tell his mother what he wants’ VICD_FGD3)* *‘communicating with your child means that loving your child to the fullest by providing things for her like…still breastfeeding her she should be able to do so in her appropriate time and the mother has to tell her that she is breastfeeding her and sometimes as a parent you have to find time to chat with the child, she can sit on your lap and talk.’ (VICD_IDI_15.)*	*’when I prepare food that she does not want before asking her what she wants to eat that day then I know that there was no communication between us, I was supposed to ask her first on what she wants to eat’ (VICD_IDI_6).*

#### Environmental safety and child protection –

Many carers specifically mentioned concerns regarding unsafe environments as a barrier to play for their child. This is particularly so when carers have other responsibilities; ‘*even for journeys you cannot leave home for long.., you are not free because you think…that if I go will I find the child alright? For s/he is blind she might fall onto fire,… be hit by a car or even a bike, as a parent you are tied up’. (VICD_FGD5.)*

Carers expressed their concern about physical or sexual abuse*; ‘It's because she is still a child who is visually impaired… I can't leave her behind for fear of people mistreating her’ (VICD_IDI_15); ‘Sometimes a child may sense some danger … and becomes hurt. Sometimes a girl child who is a bit older may be raped’. (VICD FGD5).*

## Discussion

This study describes the facilitators and barriers to enable support for young children with VI and their families. The themes emerging include barriers and facilitators not just at the level of the individual child and carer (microsystem) level, but also at the mesosystem and the exosystem where the community response and support from well‐trained professionals is vital. Table 3 outlines how these key messages and themes will be taken into account when creating stimulation programmes for children with VI in the future.

**Table 3:** Key messages for a future training programme on Care for Child Development for Children with VI

Focus	Sub‐themes	Considerations for programme	Considerations for future implementation
The Child	Additional complex needs of children with VI	High percentage of children with VI has an additional impairment.	Linkages with other intervention programmes, for example, cerebral palsy training
	Specific needs of children with VI	Need for specific advice about why children with VI can be encouraged to and how to best support them.	Programme to include specific advice on how to care and support children with VI
	Specific medical needs of children with VI	Referral criteria in programme for children with medical needs.	Clear pathways of referral for children with VI who have medical needs. Linkage between ophthalmology, skin clinic (albinism) and paediatric services and specialist teachers of VI.
	Specific difficulties with communication and socio‐emotional development	Advice in package for carers on how best to respond to their children's needs through improving methods of communication, behaviour and self‐control.	Outcome and evaluation of programmes linked to improved communication and behaviour in children.
The Family	Carer expectations for child	Advice that children with VI can develop if encouraged to play, communicate and interact.	Regular support for carers through home visitation and carefully supervised integration into Community Based Child Care Centres (CBCCs).
	Lack of knowledge or understanding on utility of play and child development	Advice that joint interaction and shared discovery through play, communication and interaction can encourage development.	Regular support through a programme scaled up with home visitation and integration Into CBCCs. Evaluation of programmes to include assessment of knowledge of caregivers on utility of play and communication for child development.
	Carer well‐being, responsibilities, need for food and concerns about child protection	Group support as well as home visitation to be used as a method for improving carer well‐being. Draw on CBCCs as a method for enabling caregivers to engage in a day‐to‐day responsibilities whilst the child is safe and being stimulated.	Evaluation of programme to include assessment of carer well‐being. Evaluation of the use of CBCCs as a model for providing respite for carers. Measurement in future use of CBCCs by carers and shared responsibility of fulfilling specific child development and learning goals.
Professionals	Lack of knowledge by professionals	Training on the UNICEF Care for Child Development VI training package.	Training on the UNICEF Care for Child Development VI training package to be integrated into training for CBCC workers and other supporting professionals (e.g. specialist teachers for VI) in Malawi.
	Lack of joined up training and services	Training and dissemination for workers in different cadres, for example, health workers within paediatrics and VI services, specialist teachers and CBR workers	Dissemination through training colleges for specialist teachers of children VI, College of Medicine and CBR training programmes.
Community	Carers and caregivers	Empowering carers to seek health, (re)habilitation and education services for their children	CBR programmes providing more information on early child development options for children and carers of children with disabilities
	Community carers	Training in CBCCs and home visits engages community carers	Training programmes provided through Ministry in country.
	Safety and independence	Training CBCC workers and specialist teachers of children with VI to encourage enabling of independence in children with VI.	Training programmes provided through Ministry in country.
	Social stigma – expectations	Training on rights‐based approaches to disability and on the rights of people with disabilities.	Anti‐discrimination programmes through the Federation of Disabled Persons of Malawi and through Malawi Council for the Handicapped.
Policy	Lack of inclusion and rights	Integration of advice for carers of children with VI into UNICEF Care for Child Development and training on rights based approaches. Refer to the United Nations Convention for the Rights of Persons with Disabilities (UNCRPD).	Integration into mainstream ECD programmes nationally (Ministry in country)and internationally (UNICEF and World Health Organisation)

ECD, early childhood development; VI, visual impairment.

In order to encourage enabling environments for children with VI to play, both the child and the carer needs to feel confident and comfortable. A strong stable microsystem can enable the child to feel confident in terms of what they are hearing and touching before they can reach out from their own personal space and explore the environment (Webster & Roe 1998; Brambring 2007). We describe in our results how caregivers naturally adapt their forms of communication and interaction to the child particularly through touch and audio description. Making sure that parents are encouraged for their already good practices is vital.

Almost all carers naturally care for their child's basic needs with many parents conceptualising the care that they give as primarily benefiting the child's health and therefore the child's ability to play.

It is clear that the mesosystem (the immediate family as well as other caregivers) and the exosystem (community workers and professional structures) are crucial for supporting families to nurture and encourage their young children with VI. Carers however, need to feel empowered to be able to support their children with disabilities (Yousafzai *et al.* 2011). To do this requires basic resources (food security and shelter) and positive mental health. Carers' lives are busy and daily family routines (carrying water, cleaning, cooking and farming) can be burdensome. Interventions, no matter how well they are designed, will have little or no impact unless wider issues at the level of the exosystem and macrosystem are challenged. A programme which advocates regular visits from support staff, with expectations that carers provide more time for their child with VI, could be disruptive on existing family arrangements. It could even increase parental stress unless it also addresses support needs of caregivers in managing their responsibilities. Our research has also shown how beneficial the provision of safe environments for children with VI and other disabilities might be.

Many carers felt the need to understand the medical condition of their child as well as the impact the condition could have on the child's ability to learn. Many children in our study also had other impairments such as cerebral palsy, social communication difficulties and hearing difficulties. Our study demonstrated that training for community workers must include basic medical information for children with a range of disabilities.

Our study has demonstrated that there are obvious influences acting on the development of the child with visual impairment at and between all levels of the ecological system. Training requirements are necessary at all levels in order to produce meaningful and appropriate programmes to support nutrition, care and early stimulation for children with VI and associated disabilities in this and similar African settings.

### Study limitations

We undertook interviews with caregivers identified through formalised channels including those presenting to ophthalmology or those with linkages to itinerant teachers. This may have influenced the views of these caregivers and the way that they manage their children. Families who had never identified themselves to services did not participate in this study and may have different views. J. C. and H. M. conducted almost all IDIs, FGDs and observations. Despite utilising triangulation and quality control at each stage, their positionality as special needs educators may have influenced the results. Furthermore, the positionality of the rest of the research team, many who work in inclusion and special needs education or medicine with experience both in Africa and in the UK may have influenced interpretation of results.

Our future research focus will seek now to understand the feasibility of undertaking adapted child development training packages to encourage stimulation for children with visual impairment by community workers in rural and urban areas of Malawi and will seek to address issues at each level of the ecological systems demonstrated in our study.

## Conclusion

This study has revealed issues to be addressed at all levels of the ecological system in order to produce meaningful and appropriate programmes to support nutrition, care and early stimulation for children with visual impairment in this African setting. The data suggest PCs require support at multiple levels. We need to aim to promote community structures, which can be strengthened to help create environments that enable both parents and children with visual impairment to enjoy greater interactive play and communication. Without taking this into consideration, programmes will not effectively support the needs of caregivers within their wider community and cultural framework.

**Key Messages**

- Visual impairment in children in low income countries is still highly prevalent
- The global community has placed emphasis on improving early child development on a global scale, but interventions do not necessarily include children with disabilities.
- We know that children with visual impairment can suffer from developmental delay and that interventions can support children with VI in terms of their development in high‐income settings, but we do not know what is appropriate for use in African settings.
- There are facilitators and barriers for the support of children with VI at the level of the individual child and family which can be addressed through programmes which encourage early child development adapted for use in children with VI.
- Encouraging children with VI to thrive and develop in African settings such as Malawi will require integration of children into community‐based early child‐care programmes if families are to feel enabled to support their children with VI.

## Conflict of Interest

There is no conflict of interest.
